# Advances in Cerebrovascular Imaging and Interventions

**DOI:** 10.3390/diagnostics15081045

**Published:** 2025-04-20

**Authors:** Seyedmehdi Payabvash

**Affiliations:** Department of Radiology, Columbia University Medical Center, New York, NY 10032, USA; sp4479@cumc.columbia.edu

Since the 1990s, the global burden of cerebrovascular disease – particularly stroke – has significantly increased [1]. According to the Global Burden of Disease (GBD) analysis, the prevalence of cardiovascular disease, including cerebrovascular disease, almost doubled from 271 million in 1990 to 523 million in 2019 [2]. While age-standardized cardiovascular mortality declined in the 1990s, the trend slowed and then plateaued in the 2000s, and since 2010, cardiovascular mortality rates have increased in many countries, including the United States, the United Kingdom, and Mexico [1]. Stroke remains the second leading cause of death globally, and the stroke-related disability-adjusted life-years (DALYs), mortality, and treatment costs are expected to double from 2020 to 2050 [1]. These trends underscore the pressing need for advancing the diagnosis, treatment and recovery of cerebrovascular disease. Advancements in neuroimaging technologies, artificial intelligence, and image-guided interventions can improve care in patients with cerebrovascular disease. This Special Issue of *Diagnostics* includes cutting-edge research reports advanced in cerebrovascular disease imaging and treatment interventions, highlighting the progress being made to improve diagnosis, therapy, and patient care.

## 1. Advanced Imaging Techniques

Neuroimaging plays a critical role in the diagnosis, management, and treatment of cerebrovascular diseases. Over recent decades, advances such as improved MRI pulse sequences and the use of intravenous contrast agents have significantly improved the capabilities of neuroimaging and deepened our understanding of the brain’s structural and functional anatomy, expanding its value in the clinical care of patients with cerebrovascular diseases. For example, in this issue, Zerweck et al. showed that an MRI-Based Assessment of Risk for Stroke in Moyamoya Angiopathy (MARS-MMA) system, including impaired breath-hold functional MRI and arterial wall contrast enhancement, is associated with impaired [15O] water PET cerebral perfusion reserve capacity. They propose that such an MRI scoring system might be a promising tool to predict the risk of stroke in Moyamoya Angiopathy [3]. The study by Chauvet et al. highlights the potential of non-contrast-enhanced 4D MR angiography as a diagnostic tool for vascular malformation in the brain with comparable accuracy to contrast-enhanced 4D MR angiography [4]. Dr. Kulcsar’s team showed the potential role of thrombotic clot dynamic perviousness, which is a measure of clot CT attenuation at multiple timepoints after intravenous contrast administration to predict clot tissue composition [5] and revascularization rate after mechanical thrombectomy in large vessel occlusion stroke [6]. And brain MR spectroscopy in patients with aneurysmal subarachnoid hemorrhage showed lower cerebral magnesium and higher pH levels in patients who developed clinically significant vasospasm.

## 2. Machine Learning and Omics Analysis

Over the past decade, machine learning and omics analysis have transformed the landscape of cerebrovascular disease by enabling more precise, data-driven approaches to diagnosis and treatment. Omics analysis, including genomics, proteomics, radiomics, and metabolomics, facilitates the identification of novel biomarkers and individualized risk profiles in cerebrovascular disease. In this issue, Avery et al. reported the prognostic role of machine learning radiomics signature of admission CT angiography scans in the prediction of collateral status and outcome in large vessel occlusion stroke [7], and Zaman et al. showed that radiomics of cerebral hematoma on admission head CT of hemorrhagic stroke patients can provide more accurate survival prediction than benchmark risk scores [8]. A combination of imaging and clinical factors in multi-modal models can provide prognostic information and guide treatment in cerebrovascular disease [9,10].

## 3. Interventional Innovations

Recent advances in neurovascular intervention have significantly improved the management and outcomes of cerebrovascular diseases, including acute ischemic stroke, cerebral hemorrhage, and aneurysms. The development of next-generation stent retrievers, aspiration catheters, and flow diverters has enhanced the safety, efficacy, and speed of endovascular treatments. Mechanical thrombectomy has become the standard of care for large vessel occlusions, with extended eligibility for large infarct core infarcts in recent trials [11]. Recent trials also showed novel clinical applications of endovascular embolization in the treatment of chronic subdural hematoma, traditionally managed with surgical evacuation with high recurrence rates and complications. The EMBOLISE trial demonstrated that middle meningeal artery embolization is a safe and effective adjunct or alternative to surgery, significantly reducing hematoma recurrence and the need for repeat interventions [12]. Studies in this Special Issue also reported recent advancements and results of intervention and results of carotid artery stenting, [13] mechanical thrombectomy [14], cerebral aneurysm coiling [15], and endovascular treatment of vertebro-vertebral arteriovenous fistula [16]. Development of novel devices, improvement of endovascular surgical techniques, and post-treatment care have enhanced the precision and safety of interventions in cerebrovascular disease. These innovations, combined with real-time imaging guidance, have minimized procedural risks and improved patient outcomes.

## 4. Future Directions and Challenges

While the advancements highlighted in this issue are promising, several challenges remain. The integration of multimodal imaging data into cohesive diagnostic and treatment frameworks requires standardized protocols and collaborative efforts across disciplines. Additionally, ensuring equitable access to cutting-edge technologies is essential to prevent disparities in care. Future research should focus on longitudinal studies that assess the long-term impact of these innovations on patient outcomes. Furthermore, the ethical implications of artificial intelligence in clinical decision-making warrant careful consideration, ensuring that technological advancements augment clinician expertise.

## 5. Conclusions

This Special Issue in *Diagnostics* highlights some of the recent innovations in cerebrovascular imaging and interventions. Applications of new imaging modalities, the use of computer vision and data science analytics, and improved surgical devices and techniques can advance cerebrovascular patient care. Novel MRI technologies, quantitative neuroimage analysis, image-guided interventions, and evidence-based treatments have the potential to improve the diagnosis, therapy, and recovery of patients with cerebrovascular disease.
